# Focus-Induced Photoresponse: a novel way to measure distances with photodetectors

**DOI:** 10.1038/s41598-018-27475-1

**Published:** 2018-06-15

**Authors:** Oili Pekkola, Christoph Lungenschmied, Peter Fejes, Anke Handreck, Wilfried Hermes, Stephan Irle, Christian Lennartz, Christian Schildknecht, Peter Schillen, Patrick Schindler, Robert Send, Sebastian Valouch, Erwin Thiel, Ingmar Bruder

**Affiliations:** 1 TrinamiX GmbH – a subsidiary of BASF SE, Industriestr. 35, 67063 Ludwigshafen, Germany; 2 ERT Optik Dr. Thiel GmbH, Donnersbergweg 1, 67059 Ludwigshafen, Germany

## Abstract

We present the Focus-Induced Photoresponse (FIP) technique, a novel approach to optical distance measurement. It takes advantage of a universally-observed phenomenon in photodetector devices, an irradiance-dependent responsivity. This means that the output from a sensor is not only dependent on the total flux of incident photons, but also on the size of the area in which they fall. If probe light from an object is cast on the detector through a lens, the sensor response depends on how far in or out of focus the object is. We call this the FIP effect. Here we demonstrate how to use the FIP effect to measure the distance to that object. We show that the FIP technique works with different sensor types and materials, as well as visible and near infrared light. The FIP technique operates on a working principle, which is fundamentally different from all established distance measurement methods and hence offers a way to overcome some of their limitations. FIP enables fast optical distance measurements with a simple single-pixel detector layout and minimal computational power. It allows for measurements that are robust to ambient light even outside the wavelength range accessible with silicon.

## Introduction

Optical distance measurement is already key to diverse applications throughout a wide range of industries. In the coming years, it is expected to gain even more importance due to the emergence of disruptive technologies such as machine vision and autonomous driving. These technologies have the power to revolutionize the world around us. However, in order to do so, further advances in optical depth sensing are required^1,2^.

**Figure 1:**
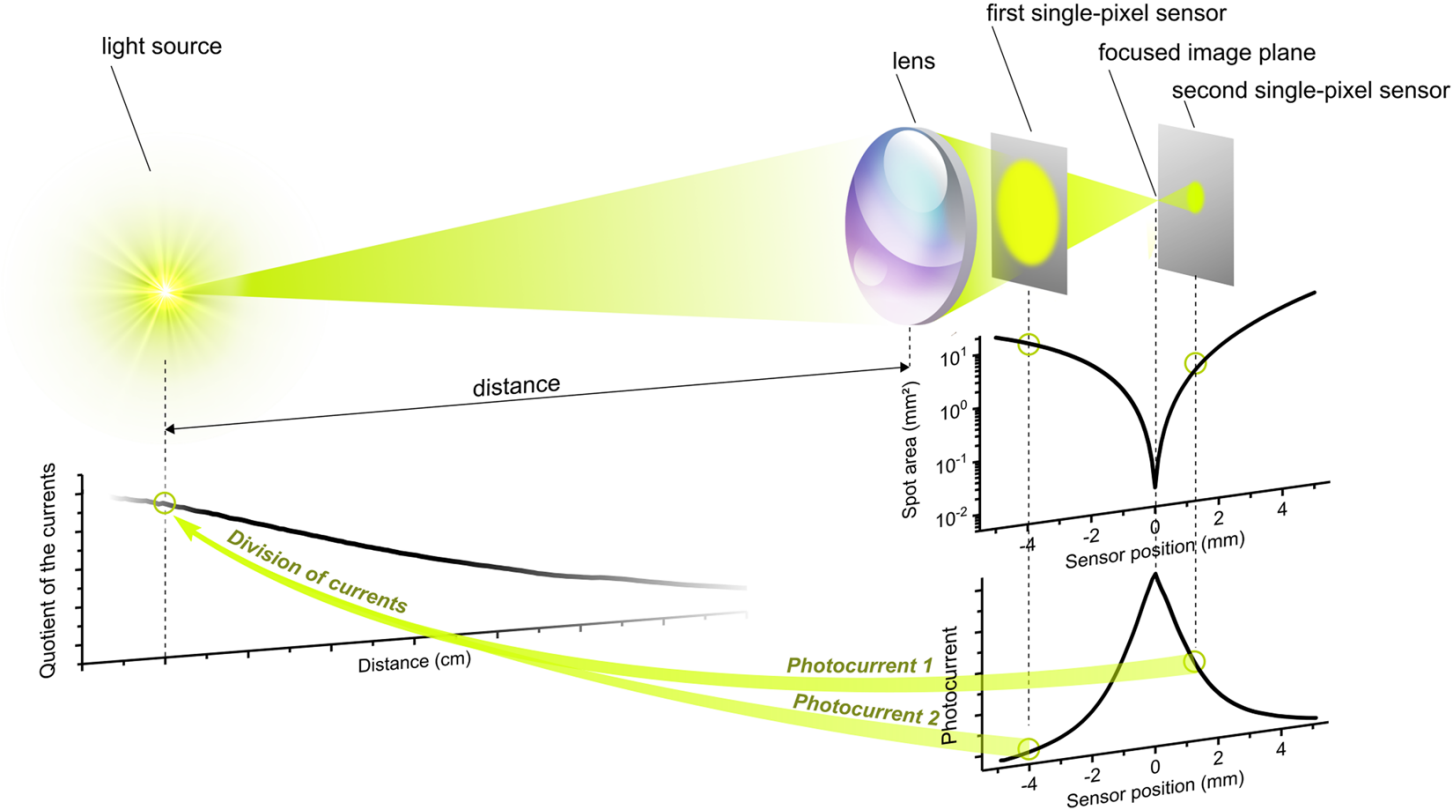
A typical setup for measuring distances with the FIP technique. For a given distance of the light source to the lens, the size of the light spot on the detector and therefore its irradiance depends on the position of the sensor behind the lens. The photocurrent of the sensor depends on the irradiance, yielding its maximum when the LED light is in focus on its active area.

Here we introduce Focus-Induced Photoresponse (FIP), a novel method to measure distances. In a FIP-based system, distance is determined directly from the analog photoresponse of a single pixel photodetector. The requirement for photodetectors to be used in the FIP technique is that their responsivity is dependent on irradiance, the incident radiant power per illuminated area. Consequently, if light is focused by a lens on a small part of the detector, its photoresponse can be distinguished from a situation when the same amount of light is spread over a larger part of the detector area. We call this the *FIP effect* and use it to measure distance (Fig. 1).

This working principle is fundamentally different from any established technique such as time-of-flight (ToF), triangulation or image-based depth sensing methods. FIP constitutes a disruptive innovation in distance measurement. It hence offers a variety of new ways to overcome intrinsic limitations of state-of-the-art techniques, thereby addressing critical challenges in the field of distance measurement.

FIP is a monocular technique, hence distance determination is possible from a single point of view. This makes the baseline calibration needed for triangulation methods unnecessary. The positions of the light source and the sensor become independent of each other. It also avoids the computationally costly solution of the correspondence problem, which is essential for stereo vision.

The FIP technique does not require taking images. Avoiding the time-consuming readout and processing of image data, FIP enables fast distance measurements. Its resolution is not limited by pixel size. The analog nature of the signal allows modulation of the light source, increasing the robustness of FIP to ambient light and direct sunlight (see Supplementary Information S1).

Methods like time-of-flight (ToF) and image-based depth sensing rely on advanced manufacturing techniques such as highly developed lithography processes and CMOS technologies^3^. FIP detectors, on the other hand, are always single-pixel devices. This makes the adaptation of other materials than silicon to the manufacturing processes technically and economically attractive. Absorber with improved sensitivity in the IR regime offer significant advantages in terms of eye safety^4^, night vision, and visibility in foggy conditions or through smoke^5^.

## Irradiance-Dependent Responsivity of Photodetectors

Irradiance-dependence is a universal phenomenon among photodetector technologies operating from the UV to the IR regime, making them suitable for the FIP technique^6–8^. The responsivity can change at low or high irradiance levels, depending on the detector. Such phenomena are observed as nonlinear relations between measured photocurrent and incident radiant power. A nonlinear photoresponse at low irradiance levels has been reported for many thin-film solar cell technologies. Trapping of charge carriers as well as photoconductivity have been identified to decrease the responsivity at low light levels^9,10^. One such example is dye-sensitized solar cells (DSSC)^11–13^. In this paper, we measure the FIP effect in solid-state DSSC and demonstrate how to use it for distance determination. Time-resolved photocurrent experiments reveal the difference in performance when the same amount of light is in or out-of-focus on the solar cell.

On the other hand, high light intensities exceeding 1 sun can reduce the current collection in solar cells, decreasing the conversion efficiency of these devices at high irradiances^6,14^. The reduced photovoltaic performance has been attributed to a change in recombination mechanisms and a change in series resistance with irradiance^14^. In organic solar cells, recombination has been found to depend on the charge carrier concentration^15–17^.

In addition to photodiodes, also photoconductors show an irradiance-dependent responsivity. Using an experimental setup similar to that of the FIP technique, a reduced photoresponse in PbS and HgCdTe photoconductors has been reported when only a small area of the device receives high-intensity illumination^7,8,18^. In this publication, we show that photoconductors can be used as FIP detectors. We demonstrate the FIP effect on PbS photoconductors using 1,550 nm illumination, making use of their ability to absorb wavelengths deeper in the IR than the absorption spectrum of silicon. The responsivity of PbS is demonstrated to reduce when the same amount of light is focused on a smaller part of the detector area. We model the partially illuminated photoconductors as a network of light-dependent resistors. We show that even when the photoresponse of a photoconductor depends linearly on irradiance, a FIP effect is observed when the active area is only partly illuminated.

## Results

### The FIP effect in dye-sensitized solar cells (DSSC)

In DSSC, the dependence of photovoltaic performance on light intensity is published in detail^11–13^. A reduced conversion efficiency is reported for low intensities of modulated light. DSSC contain a mesoporous (mp) TiO_2_ layer sensitized with dye molecules. mp-TiO_2_ acts as the electron transporting material, and its pores are filled with a hole transporter. In the experiments presented below, the hole transporting layer is made of a solid film of the organic material spiro-MeOTAD^19^; the device is hence referred to as a solid-state DSSC (sDSSC). Charge transport in the mp-TiO_2_ structure is strongly impeded by localized states in the band gap. These traps are occupied by photogenerated electrons which can be thermally de-trapped to the conduction band, where they are mobile. The trapping/de-trapping model^20^ explains the experimental finding that the electron diffusion coefficient in the mp-TiO_2_ increases with the electron concentration^11,12,21,22^. A higher density of absorbed photons increases the Fermi level and more electrons are de-trapped leading to faster electron transport.

**Figure 2:**
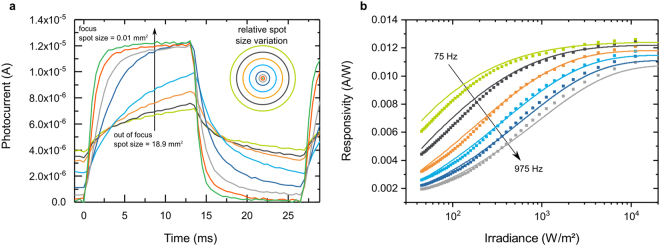
Irradiance-dependent responsivity of sDSSC. (**a**) Transient photocurrent response of a sDSSC to pulsed illumination through a lens. The position of the device on the optical axis is varied, hence the same incident radiant power is distributed over different surface areas. The relative variation of the light spot sizes is illustrated by the circles. (**b**) Responsivity of a sDSSC as a function of the irradiance for different modulation frequencies. The scatter points assume uniform illumination of the light spot. The solid lines are based on a ray-tracing model.

The effect can be seen in the photocurrent transients shown in Fig. 2a. The sDSSC sample was illuminated through a lens by a square wave modulated 530 nm LED. While the distance between the LED and the lens was kept constant, the spot area was varied between 0.01 mm^2^ and 19 mm^2^ by moving the solar cell along the optical axis around the focused image plane. Assuming a uniform light distribution, this corresponds to irradiances between 25–6,000 W/m^2^ (for details about the spot size and irradiance calculations, see Supplementary Informations S2 and S3). We observe that increasing the irradiance strongly shortens the rise time of the photocurrent. At the two highest irradiance levels shown in Fig. 2a, the identical equilibrium photocurrent is reached within the pulse period and both currents reduce to zero within the dark period. All measured transients at lower irradiance levels are already too slow to reach equilibrium or decay to zero within the pulse duration. We interpret this behavior as a direct consequence of the reported increase of the diffusion coefficient of the electrons in mp-TiO_2_ with irradiance.

Figure 2b shows the responsivity of the sDSSC as a function of the irradiance at modulation frequencies between 75 Hz and 975 Hz. Irradiances between 10 and 10,000 W/m^2^ were covered by moving the sDSSC behind the lens in 0.1 mm steps and thereby changing the size of the image on the sensor. The scatter points represent the photocurrent densities measured at each position. As in Fig. 2a, the areas of the light spots were calculated with paraxial optics and assumed to be uniformly illuminated (see Supplementary Information S2). To test the validity of this assumption, we also obtained beam profiles by ray-tracing and used a segmentation approach to parametrize the photocurrent densities as a function of the irradiance. The model is described in detail in Supplementary Information S4. The resulting responsivities are plotted as lines in Fig. 2b. We find good agreement between the modeled responsivity and the experimental data, which assumes uniformly illuminated light spots. The deviation of the responsivity from the plateau observed at high irradiance levels increases with modulation frequency. The alternating photocurrent induced by a modulated light source can thus be larger at higher photon densities even though the total amount of light on the sample stays constant: the solar cell works more efficiently at high irradiance. This is the signature of the FIP effect in sDSSC.

### Measuring distances with the FIP technique

In the following, we demonstrate how the FIP effect can be utilized for distance measurements with a setup schematically depicted in Fig. 1. Its components are a modulated LED (or any light source that either actively emits or reflects light), a consumer grade camera lens (or any optical element that captures the light and focuses it), two photodetectors, and a signal processing unit. The lens collects and focuses the light of the LED. The semitransparent sensors are placed behind the lens near its focal plane. The modulated light of the LED generates an alternating photoresponse, which is then amplified and recorded using lock-in or Fourier transform techniques. The spot size on the sensor changes with the distance between the light source and the lens as the position of the focused image plane shifts. The active area must therefore be large enough to accommodate the maximum spot size within the desired measurement range.

There are two main contributions to the sensor output. The first is due to the FIP effect: when the modulated light source is positioned at a certain distance from the lens, the measured photocurrent depends of the irradiance on the sensor, i.e. how well the light is focused. The strong focusing of the light can change the size of the light spot and thus the irradiance on the sensor for several orders of magnitude, which leads to an appreciable change in responsivity of the irradiance-dependent sensor. Secondly, the photocurrent is impacted by the total amount of light collected by the lens. When the LED is moved away from the lens, this contribution decreases. If the radiant power of the light source is unknown, the photoresponse of a single detector at any given position behind the lens does not allow for an unambiguous distance determination. Whether the LED is distant and bright or close and dim cannot be distinguished. To solve this problem, we use two detectors in the beam path, see below. By calculating the ratio of the two photoresponses, the distance dependence as well as fluctuations in the output of the light source cancel out. Due to the FIP effect, the quotient changes with the distance, yielding a unique signature for each LED position.

### Demonstrating the FIP technique with sDSSC

**Figure 3:**
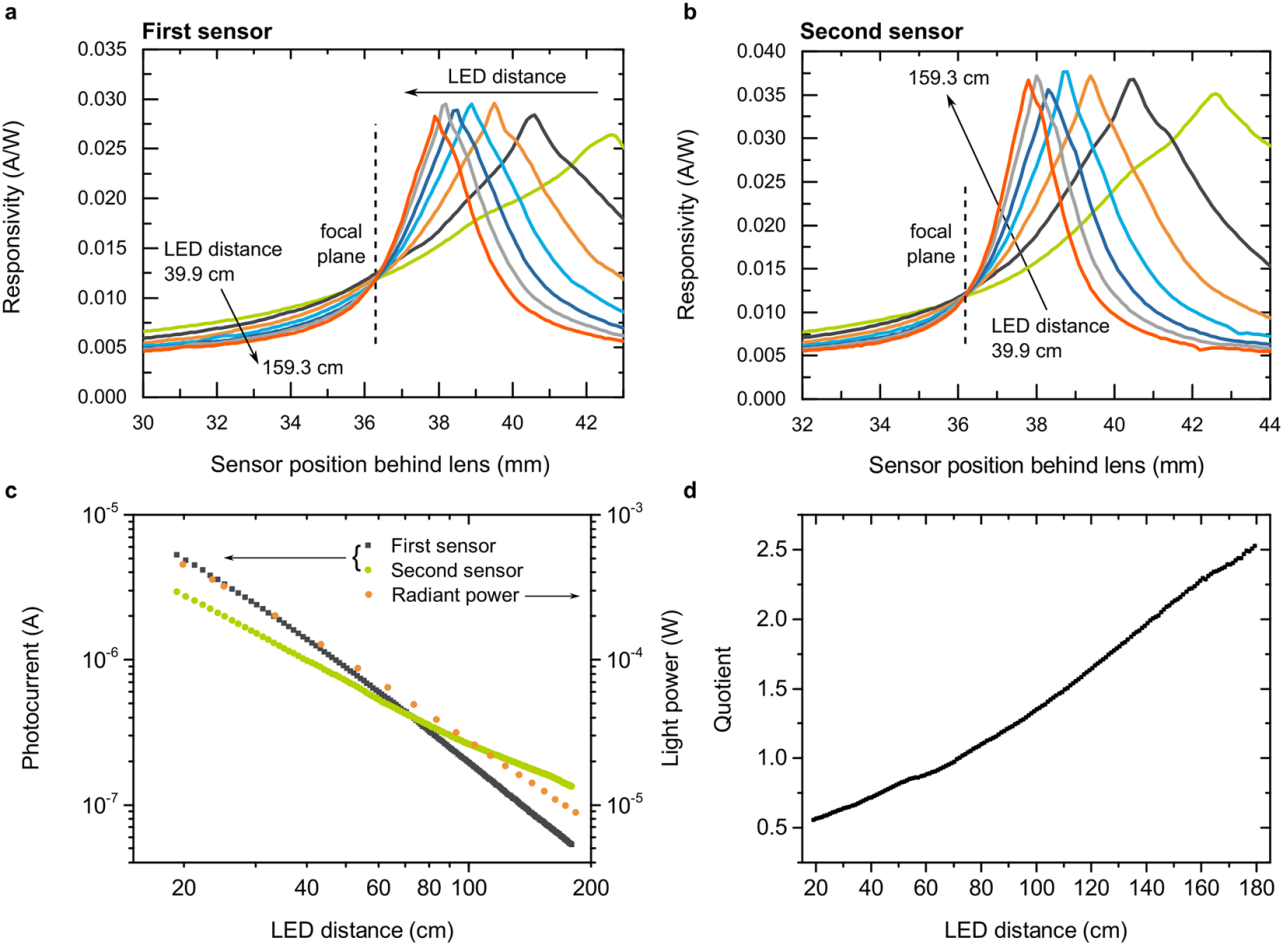
Distance measurement with sDSSC. (**a**,**b**) Responsivity of the first (**a**) and second (**b**) sDSSC sensor for a range of positions behind the lens at various LED distances. The second sensor is illuminated through the first one. (**c**) The absolute photocurrent of both sensors and the radiant power on the first sensor as a function of LED distance. (**d**) The quotient of the photocurrents as a function of LED distance.

We have realized such a setup by using two semitransparent sDSSC as sensors and a modulated green LED. The results are summarized in Fig. 3. Figure 3a and b show the responsivities of both sDSSC as a function of their position behind the lens. The responsivity was calculated by normalizing the short circuit current to the total radiant power incident on the device. The sensor closer to the lens is referred to as the first sensor, and the one further from the lens as the second sensor. Due to the FIP effect, the maximum photocurrent is recorded when the sensor is positioned in the focused image plane. In this case, the LED light is focused on the sensor. A reduction in the current is observed as the light spot widens symmetrically. With increasing LED distance, the focused image plane moves towards the focal plane of the lens, leading to a shift of the photocurrent peaks. As shown in Fig. 3a and b, we observe that the responsivity curves intersect at the focal plane of the lens. In this point, the irradiance in the light spot is independent of the LED distance (for the derivation, see Supplementary Information Section S5). This observation is consistent with our assumption that the FIP effect is induced by the irradiance-dependent sensor responsivity.

The positions of the sensors in the beam path may be adjusted to the specific measurement problem. In the presented case, we measure distances in the range between 20 cm and 1.8 m (Fig. 3c and d). The first sensor is positioned 33.3 mm behind the lens, between the lens and its focal plane. The second sensor is placed 37.6 mm behind the lens, between the focal plane and the closest responsivity maximum. With increasing LED distance, the responsivity of the first sensor decreases, while it increases for the second sensor. Therefore, a large change in the ratio of their photocurrents is achieved over the measurement range. The absolute photocurrents of both sensors as well as the incident radiant power are plotted in Fig. 3c. The photocurrents are dominated by the inverse dependence of the radiant power on LED distance. The non-equilibrium photocurrents deviate from linearity. This behavior is visible as a difference in slopes. The resulting quotient of the photocurrents increases monotonically over the entire measurement range (Fig. 3d), assigning a single value to each object distance. With this calibration curve, the LED distance can be directly determined by simply measuring the individual photocurrents of both cells and calculating their ratio.

### The FIP effect in PbS photoconductors

As described above, the reduced responsivity observed in DSSC at low irradiance and sufficiently large frequencies causes a maximum photoresponse when the device is placed in the focused image plane. In this situation, the irradiance and consequently the responsivity of the device are maximized. For other thin-film solar cell and photoconductor technologies, a reduced photoresponse has been reported when only a small area of the device receives high intensity illumination^6–8^. Due to the resulting nonlinear photoresponse at high incident power, we expect the photoresponse of these devices to reach a minimum when positioned in the focused image plane. PbS photoconductors used as IR sensors are an example of such behavior. In contrast to photovoltaic detectors, photoconductors do not generate a photocurrent. Instead, their resistance changes upon illumination. Even though the functionality differs fundamentally from DSSC, these devices can be used for distance measurements with the FIP technique. However, PbS detectors are opaque and cannot therefore be used in sensor stacks like the sDSSC presented above. Instead, a beam splitter may be used to position both sensors at appropriate distances from the lens.

**Figure 4:**
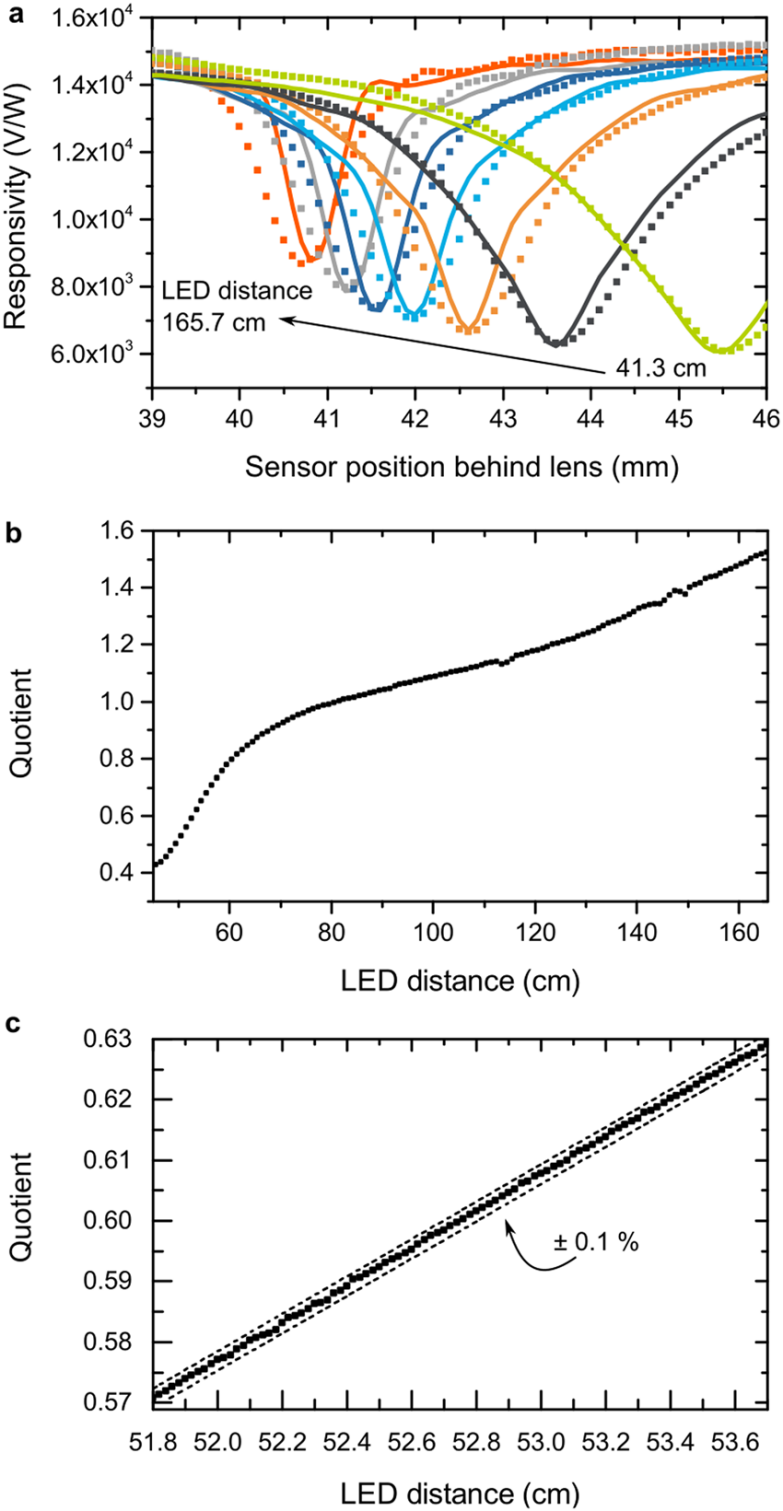
Distance measurement with PbS. (**a**) Responsivity of a PbS photoconductor for a range of positions behind the lens at various LED distances. The device is modeled as a network of infinitesimally small photoconductors each showing a linear response to irradiance. The lines represent best fits to the experimental data. (**b**) The quotient of the photoresponse of the PbS device at 40.4 mm and at 45 mm as a function of LED distance. (**c**) detail of the quotient curve with an indication of a corridor of ±0.1% of the distance to illustrate the obtained resolution.

Figure 4a shows the responsivity of a PbS detector to modulated 1,550 nm illumination as a function of its position behind the lens for various LED distances. The photoresponse is recorded as a voltage using an amplifier circuit. We determine the responsivity by normalizing the photoresponse to the radiant power reaching the detector. A minimum in the responsivity is observed when the light spot is focused on the detector.

To understand this behavior, we have modeled the responsivity of the tested PbS photoconductor to light of various spot sizes and irradiance levels. The device is simulated as a two-dimensional grid of connected infinitesimally small photoconductor elements. By performing a limiting process, we can model the grid with an elliptic partial differential equation, i.e. $\nabla \cdot (\frac{\nabla \phi }{R(x,y)})=\nabla \cdot ({\rm{\sigma }}({\rm{x}},{\rm{y}})\nabla \phi )=0$, where φ(x, y) is the electric potential, R(x, y) the local resistance and σ(x, y) the conductance (more details in Supplementary Information S6). This model can be solved using the Finite Elements Method. The conductance *σ* of every point of the PbS photoconductor is described as *σ* = *σ*_*d*_ + Δ*σ*. It is hence composed of the dark conductance *σ*_*d*_ and a change *Δσ* due to illumination. *Δσ* is assumed to be directly proportional to the irradiance $(E\sim \frac{W}{{m}^{2}})$, i.e. Δ*σ* = *p* · *E*. The irradiance is calculated using paraxial optics and assumes uniformly illuminated light spots (see S2). We have fitted the measured data plotted in Fig. 4a using ${\rm{\Delta }}\sigma =0.298\,\frac{mm}{{V}^{2}}\cdot E$.

The resulting curves are shown as lines in Fig. 4a. We interpret the good agreement between the measured data and our model as a confirmation of the assumed linear dependence of Δσ on irradiance for the tested PbS photoconductor within the studied irradiance regime from 0.3 W/m^2^ to 5,000 W/m^2^. Even if a photoconductor reacts perfectly linearly to the irradiance, a FIP effect is observed when the active area is only partially illuminated. This behavior is consistent with experimental data^15^ and shown formally in Supplementary Information S6.

The FIP effect in PbS photoconductors is used for distance measurements by calculating a photoresponse quotient for each measured distance (Fig. 4b). The ratio of the photoresponses was determined for sensor positions at 45 mm and 40.4 mm behind the lens. The resulting quotient increases over the studied measurement range, enabling accurate distance measurements between 45–165 cm. Figure 4c depicts a small range to illustrate the resolution of distance measurements with the FIP technique. LED positions as close together as 500 µm can be distinguished by the photoresponse quotient at a distance of 52 cm, corresponding to a depth resolution of better than 0.1%.

## Conclusions and Outlook

We have introduced FIP, a novel distance measurement technique, which utilizes sensors with an irradiance-dependent responsivity. Irradiance-dependence is a universal phenomenon in photodetectors, which enables the utilization of different types of sensors. In this paper, the functionality of the technique was shown with sDSSC using visible light as well as PbS photoconductors using NIR illumination. Additionally, the FIP effect has been experimentally demonstrated in various thin-film photovoltaic device technologies such as amorphous silicon, CdTe, CIGS, CIS, CZTS, as well as in organic solar cells^23^. Whereas the physical phenomena causing the FIP effect in these devices may differ significantly, all of them can be used for distance measurements with the FIP technique. The wavelength of the illumination can thus be flexibly chosen for the needs of the measurement task at hand. It is possible to use wavelengths deeper in the IR than the absorption of silicon.

Measurement range and resolution are largely determined by the choice of optics, which makes FIP a very versatile technology. In this paper, we demonstrated the measurement principle at distances up to 2 m and showed a resolution of below 500 µm at a distance of 50 cm. In the Supplementary Information S7, distance measurements up to 70 m can be found.

FIP can be combined with other technologies to create systems with even more functionality. A device sensitive to the x, y and z coordinates of a light spot^24^ can be created by using commercially available position sensitive devices (PSD) as the sensors. Simultaneous tracking of multiple light spots is possible if they have different modulation frequencies. Measuring distances with a FIP detector can be done in two distinct ways, in a tracking mode or a scanning mode. In the first, the position of light-emitting markers in space is tracked. This approach is called motion tracking or motion capture. Conventional motion capture systems are dominated by stereo camera systems. Each marker needs to be visible by at least two cameras simultaneously to determine its coordinates. The camera positions require accurate calibration, then the images are read out and analyzed to identify the marker positions. The distance is then calculated by triangulation. These processes are expensive in terms of equipment as well as computational cost. The read-out of the image sensor slows down the position determination.

FIP circumvents these problems by determining distance information from the analog response of a single-pixel detector. It is hence a fast and computationally lean method that requires no image processing. The technique is robust to ambient light and requires only low computational power. Distance information is retrieved from a single sensor unit without triangulation.

The second way to use FIP is the scanning mode. It utilizes projected light spots instead of actively emitting ones. This allows lasers to be used as the light sources instead of the LEDs we have presented here. While this can be readily achieved with commercial systems using ToF or triangulation, FIP offers the advantage that the position of the laser’s origin does not impact the measurement. The laser can thus be positioned freely, because the FIP technique only measures the distance to the light spot. Laser alignment is not critical as the source can be moved without affecting the distance measurement.

In order to fully capitalize on the advantages of this fundamentally novel technique, FIP detectors will benefit from further performance optimization. With improved understanding of the underlying mechanisms and further development, FIP will solve distance measurement challenges that have been intractable with state-of-the-art techniques.

## Methods

### Fabrication of sDSSC

The FTO substrates (Pilkington glass) were first cleaned with a glass detergent, then rinsed with water and cleaned with acetone and isopropanol. Subsequently, the substrates were ozone treated for 30 min (Novascan PSD Series Digital UV Ozone System). After that, a TiO_2_ blocking layer was deposited on the substrates via spray pyrolysis. 9.72 g titanium diisopropoxide bis(acetylacetonate) was dissolved in 100 ml ethanol. 25 spray cycles were performed at 350 °C. After the pyrolysis, the samples were annealed at 350 °C for 30 min. For the deposition of a mesoporous TiO_2_ layer, transparent titania paste (Dyesol, average particle size 20 nm) was mixed with ethanol in a ratio of 1:3. The solution was spin coated at 3700 RPM for 30 s, and the films were sintered subsequently at 450 °C for 30 min. The TiO_2_ films were immersed in a 5 mM dye (N-Carboxymethyl-9-(7-(bis(9,9-dimethyl-fluoren-2-yl)amino)-9,9-dihexyl-fluoren-2-yl)perylene-3,4-dicarboximide) solution in toluene for 1 h. After that the samples were rinsed with water and dried with nitrogen. 100 mg/ml hole conductor 2,2′,7,7′-Tetrakis[N,N-di(4-methoxyphenyl)amino]-9,9′-spirobifluorene (Spiro-MeOTAD) in chlorobenzene was mixed with 20 mM bis(trifluoromethane) sulfonamide lithium salt in cyclohexanone and 2.5 mg/ml vanadium pentoxide, and oxidized in air for 1 h. Subsequently, vanadium pentoxide was removed by filtering the solution through a 0.2 µm PTFE filter. The solution was spin coated at 2000 RPM for 30 s and the samples were left to dry for 30 min. We used PEDOT:PSS (Clevios F HC Solar) as the counter electrode. The dispersion was filtered with a 0.45 µm PTFE filter and spin coated at 2000 RPM for 30 s. After that, the samples were dried on a hot plate at 90 °C. Finally, 200 nm Ag contacts were evaporated on top of the PEDOT:PSS film using a custom-made Creavac thermal evaporator. The transmittance of the sensors was 42.3% at 530 nm.

### Transient photocurrent

The sDSSC was illuminated with a 530 nm LED (Thorlabs M530L3). The LED was modulated at 375 Hz with square wave pulses and a duty cycle of 50%. The light was focused with an aspheric lens (Thorlabs AL2520-A) that was positioned at 35.2 mm from the LED. The sensor was mounted on a translational stage that allowed movements along the optical axis. The light power on the sensor was 465 µW, and the size of the image on the sensor was changed by moving the sensor on the optical axis. The transient photocurrent was amplified with a Femto DLPCA transimpedance amplifier (gain 10^4^ V/A) and recorded with a National Instruments PXIe-4492 measurement card.

### Photocurrent as a function of photon density and modulation frequency

The measurement setup was identical to that of transient photocurrent as described above. The alternating photocurrent was amplified with a Femto DLPCA transimpedance amplifier (gain 10^4^ V/A) and recorded with a Behringer U-Phoria UMC202HD sound card. The LED was modulated at 75, 175, 375, 575, 775, and 975 Hz, and the light power on the sensor was 855 µW.

### FIP distance measurements with DSSC

A stack of two semitransparent sDSSC was illuminated with a square wave pulsed LED (Thorlabs M530L3) at 530 nm. The LED was modulated with square wave pulses at 475 Hz. The light was focused with a Nikkor 50 mm f/1.2 lens. The distance between the sensors in the stack was 4.3 mm. The stack was mounted on a translational stage and the LED on a rail that allowed movements along the optical axis. The radiant power on the first sensor at LED distance of 13.5 cm was 790 µW. The photocurrents were amplified with two Femto DLPCA-200 transimpedance amplifiers (gain 10^4^ V/A) and recorded with two lock-ins (SR 850, Stanford Research Systems).

### FIP distance measurements with PbS and the photoconductor model

A commercial PbS photoconductor (Hertzstück^TM^, active area 1 cm × 1 cm) was illuminated with an LED at 1,550 nm (Thorlabs M1550L3). The LED was modulated with square wave pulses at 606 Hz and its light was focused with a Nikkor 50 mm f/1.2 lens. The photoresponse was measured using a voltage divider including a 2 MΩ resistor to match the dark resistance of the PbS device of similar resistance. A voltage of 100 V was applied to the photoconductor and the 2 MΩ resistor, hence an electric field of 50 V/cm was present across the active area of the photodetector. The photoresponse to the modulated LED was then determined with a Behringer U-Phoria UMC202HD sound card connected via a unity gain buffer. The experimental data are obtained using an FFT with a bandwith of 1 Hz. This can be interpreted as a smoothing process. In order to remove numerical perturbations, the simulated results were smoothed by using a moving average filter as well. The PbS photoconductor was mounted on a translational stage and the LED on a rail that allowed their movements along the optical axis. The radiant power on the sensor at an LED distance of 13.5 cm was 35.1 µW.

## Electronic supplementary material

Supplementary Information
