# Supplementary material for: Focus-Induced Photoresponse: a novel way to measure distances with photodetectors
Source: Sci Rep. 2018 Jun 15;8:9208. doi: 10.1038/s41598-018-27475-1 (PMC6003943; doi:10.1038/s41598-018-27475-1)
Supplement: Supplementary file 1 — Supplementary Information [file 41598_2018_27475_MOESM1_ESM.pdf]

Focus-Induced Photoresponse: a novel way to measure distances with photodetectors

Authors: Oili Pekkola[1], Christoph Lungenschmied[1,*], Peter Fejes[1], Anke Handreck[1], Wilfried Hermes[1], Stephan Irle[2], Christian Lennartz[1], Christian Schildknecht[1], Peter Schillen[1], Patrick Schindler[1], Robert Send[1], Sebastian Valouch[1], Erwin Thiel[2], Ingmar Bruder[1]

Affiliations:

[1]trinamiX GmbH – a subsidiary of BASF SE, Industriestr. 35, 67063 Ludwigshafen, Germany.
[2]ERT Optik Dr. Thiel GmbH, Donnersbergweg 1, 67059 Ludwigshafen, Germany.
*Correspondence to: christoph.lungenschmied@trinamix.de

Supplementary information

S1 – FIP measurement on PbS with background illumination

Fig. S1.1 shows FIP curves of a PbS sensor measured at 1,550 nm. The sensor was illuminated with a modulated LED at 606 Hz through a Thorlabs FB1550-40 bandpass filter. The black curve corresponds to the measurement without additional background illumination. The radiant power of the LED light on the sensor was 1 µW.

To demonstrate the robustness of the measurement against white ambient light, the sensor was additionally illuminated by a construction site flood light that was placed behind the LED (the green curve in Fig. S1.1). The power of the flood light illumination at the sensor was 20 μW. As seen in Fig. S1.1, the light bias does not disturb the FIP measurement.

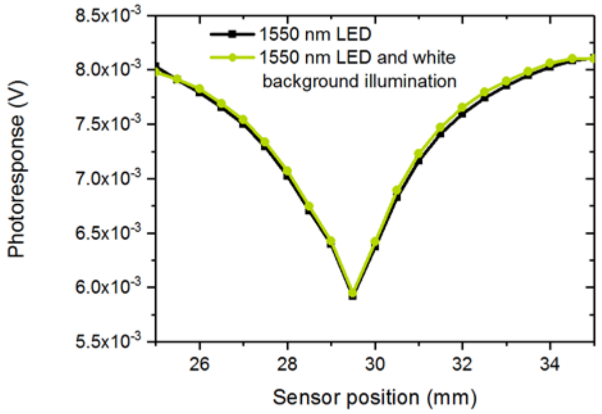

Figure S1.1 FIP curves of a PbS sensor measured at 1,550 nm through a bandpass filter. Black: without background illumination, green: with white background illumination

S2 – Modelling an optical image with paraxial optics

We use paraxial optics to estimate the size of an optical image on the FIP sensor. The optical setup is described by the paraxial approximation, i.e. the model is based on the thin lens equation

$$\frac{1}{f} = \frac{1}{z} + \frac{1}{b} \qquad (S2-1)$$

where f is the focal length of the lens, z the distance between the light source and the lens and b the distance between the lens and the focused image of the light source. It should be noted that the model is an approximation. The irradiance distribution within the image or the properties of the lens are not considered.

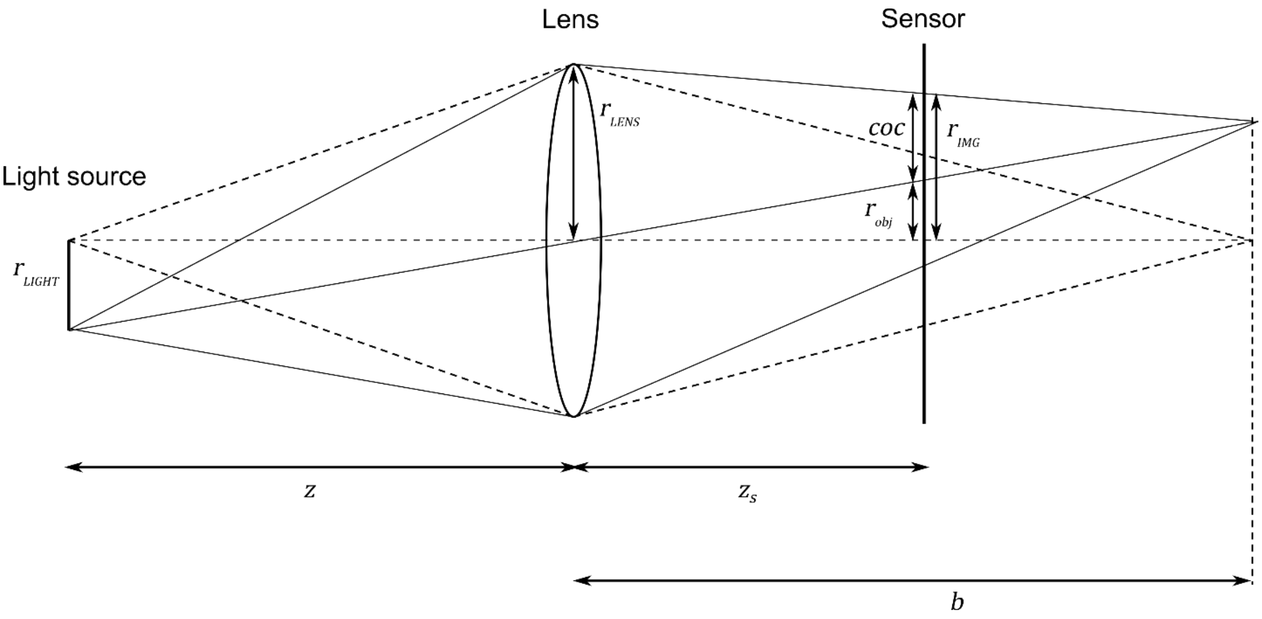

Fig. S2.1 Optical setup

The disk-shaped light source has a radius r_{LIGHT}. The sensor is placed at a distance z_s behind the lens. The image of the light source on the sensor has a radius r_{IMG}. It consists of the image of the light source without blur, r_{OBJ}, and the circle of confusion coc:

$$r_{IMG} = r_{OBJ} + |coc| \qquad (S2-2)$$

The circle of confusion is determined by the intercept theorem, i.e.

$$\frac{coc}{b - z_s} = \frac{r_{LENS}}{b}. \qquad (S2-3)$$

Combining equations S2 – 1 and S2 – 3, the circle of confusion yields to

$$coc = r_{LENS}\left(1 - \frac{z_s}{b}\right) = r_{LENS}\left(1 - z_s \cdot \left(\frac{1}{f} - \frac{1}{z}\right)\right) = r_{LENS}\left(1 - z_s \cdot \left(\frac{z - f}{fz}\right)\right). \qquad (S2-4)$$

The object size on the image r_{OBJ} is also determined by the intercept theorem, i.e.

$$\frac{r_{OBJ}}{z_s} = \frac{r_{LIGHT}}{z}. \qquad (S2-5)$$

The radius of the optical image r_{IMG} is thus

$$r_{IMG} = r_{LENS}\left|1 - z_s \cdot \left(\frac{z-f}{fz}\right)\right| + \frac{r_{LIGHT}}{z} z_s. \qquad (S2-6)$$

This formula can be extended easily by using the diameter d_{LENS} instead of the radius:

$$d_{IMG} = d_{LENS}\left|1 - z_s \cdot \left(\frac{z-f}{fz}\right)\right| + \frac{d_{LIGHT}}{z} z_s. \qquad (S2-7)$$

S3 – Image sizes and photon densities in transient photocurrent measurements (Fig. 2a)

Distance of the sensor from the focused image plane: 0.2 – 5 mm, power of the light source: 465 µW

Sizes of the light spot were calculated with paraxial optics (Eq. S2-7) with following parameters:

- Distance of the light source 35.2 mm
- Diameter of the light source 2 mm
- Focal length of the lens 20 mm
- Working F# 0.979877

Distance from focused image plane (mm)	Spot area (mm^2)	Irradiance (W/m^2)
0.2	0.08	6087.73
0.5	0.28	1650.56
1	0.91	510.09
2	3.25	143.08
3	66.18	66.18
4	12.24	37.99
5	18.90	24.61

S4 – Simulation of beam profiles with ray tracing

Although paraxial optics allows for a qualitative understanding of the imaging process, a ray tracing model accounting for actual lens systems is needed. Since the sensor signal depends on irradiance, special care must be taken to account for the spatial distribution of photons over the sensor area. It is not possible to assign a single irradiance value to a specific beam profile in a FIP measurement, since each profile on the sensor consists of a characteristic distribution of local irradiances. To deduce the specific relation between irradiance and the current density of a certain sensor type, we have chosen the following ansatz:

We discretize the sensor area with a rectangular grid. At each sensor position, every pixel is assumed to behave as a local sensor that is exposed to a discrete irradiance $E_{i,s}$. The discrete irradiance depends on the radiant power distribution Φ_{local} at that pixel and sensor position:

$$E_{i,s} = \frac{\Phi_{local}(x_i, y_i, z_s)}{A_{local}}, \qquad (S4-1)$$

where x_i and y_i are the pixel coordinates, z_s the sensor position with respect to the lens and A_{local} the area of each pixel. We now define the local response function f_{local} as

$$f_{local} = p_1 \cdot E_{i,s} - p_2 \cdot E_{i,s} \cdot \exp\left(-p_3 \cdot {E_{i,s}}^{p_4}\right), \qquad (S4-2)$$

where $p_1 - p_4$ are simulation parameters. The local response function gives the local current $I_{i,s}$ originating from the local irradiance:

$$I_{i,s} = f_{local}\left(E_{i,s}\right) \qquad (S4-3)$$

By summing over all local currents, we obtain the overall sensor response current I_s for each sensor position z_s:

$$I_s = \sum_i f_{local}\left(E_{i,s}\right) \qquad (S4-4)$$

To obtain the parameters of the local response function, we used the simulated irradiation profiles at different sensor positions in combination with the experimentally measured photocurrents and performed a least-squares fit to obtain the best matching local response function.

Specifically, we used the following parameters:

- Lens: Thorlabs AL2520M-A, Mounted Asphere, Ø25.0mm, EFL = 20.0mm, NA=0.54, -A Coating
- LED position with respect to the lens: 32.5 cm
- Wavelength: 530 nm
- Width of the discretized sensor: 7 mm
- Number of pixels per line/column: 1,001
- Number of simulation rays: 5,000,000

Parameters for different modulation frequencies:

**Frequency**	p_1	p_2	p_3	p_4
975 Hz	0.001077475772490	0.001090632227783	0.026971714492125	0.531302606764043
775 Hz	0.001112257999756	0.001151022565467	0.034556007165795	0.523592469700944
575 Hz	0.001149372503804	0.001279720508382	0.058938000077514	0.479743037095579
375 Hz	0.001181978599514	0.001493075487758	0.109307540253059	0.430996445237502
175 Hz	0.001221120774188	0.002960866589844	0.477235010400842	0.281457998864533
75 Hz	0.001242651802542	0.012728796621985	1.658609785709937	0.166377333205729

S5 - Modelling the irradiance of a photodetector through a lens

Assumptions:

- The light source is infinitesimally small (point light source) and emits uniformly in all directions
- The optical setup is described by the paraxial approximation, observing the thin lens equation S1-1
- The sensor is larger than the image of the light source

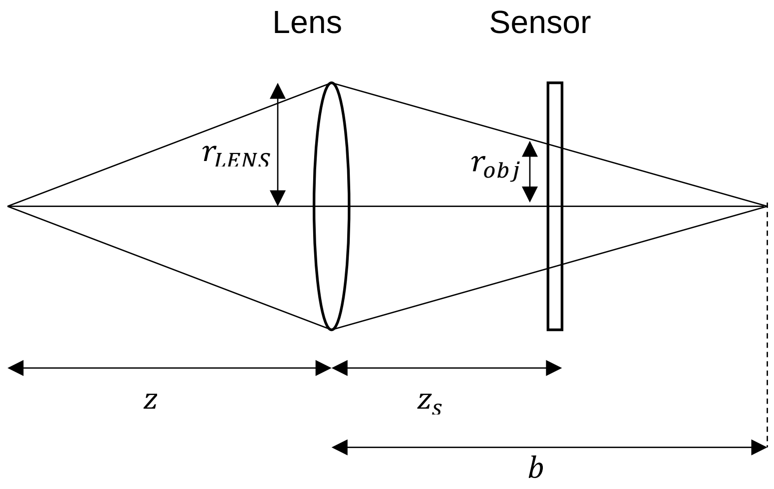

Figure S5.1: Optical Setup

In consideration of these assumptions, the optical image on the sensor is a circular disk. Its radius (r_{obj}) is given by

$$r_{obj} = \left| r_{LENS} \left(1 - z_s \frac{z - f}{zf} \right) \right|, \qquad (S5 - 1)$$

where r_{LENS} is the radius of the lens and z_s is the distance between lens and sensor.

The amount of light shining on the sensor is modeled by the radiant power L. For sufficiently large z, it decreases with the square of the distance between light source and lens. The parameter λ_0 characterizes the emitted light and the transmission properties of the lens.

$$L(z) = \frac{\lambda_0}{z^2} \qquad (S5-2)$$

The irradiance E of the sensor is given by the distribution of the radiant power on the sensor

$$E(z) = \begin{cases} \frac{1}{\pi r_{obj}^2} L(z) & \|x\| \leq r_{obj} \\ 0 & \|x\| > r_{obj} \end{cases} \qquad (S5-3)$$

The nonlinear sensor response function to the irradiance is defined by F. The overall sensor response is the spatial integral over the irradiance, i.e.

$$I(z) = \int F\big(E(x)\big)dx = \pi r_{obj}^2 \cdot F\left(\frac{1}{\pi r_{obj}^2} \cdot L(z)\right). \qquad (S5-4)$$

The normalized sensor response I_{norm} is defined by

$$I_{norm} = \frac{I(z)}{L(z)}. \qquad (S5-5)$$

Iso-FIP theorem:

Let the sensor position set to $z_s = f$. Then the following result is valid: For any sensor response function F the normalized sensor response does not depend on the distance z.

Proof:

Let $z_s = f$, the radius of the illuminated disk reduces to

$$r_{obj} = \frac{r_{LENS} \cdot f}{z}. \qquad (S5-6)$$

Then the normalized sensor response I_{norm} yields to

$$I_{norm} = \frac{\pi r_{LENS}^2 f^2}{z^2} F\left(\frac{z^2}{\pi r_{LENS}^2 f^2} L(z)\right) L(z)^{-1}. \qquad (S5-7)$$

Plug in the irradiance function on the sensor L:

$$I_{norm} = \frac{\pi r_{LENS}^2 f^2}{z^2} F\left(\frac{z^2}{\pi r_{LENS}^2 f^2} \frac{\lambda_0}{z^2}\right) \frac{z^2}{\lambda_0} = \frac{\pi r_{LENS}^2 f^2}{\lambda_0} F\left(\frac{\lambda_0}{\pi r_{LENS}^2 f^2}\right). \qquad (S5-8)$$

For the normalized sensor response, z cancels out, hence the response does not depend on the distance z.

□

Explanation:

When the distance between light source and sensor (z) increases, the amount of light impinging on the sensor decreases. At the same time, the size of the optical image decreases. Both trends contribute to the amount of light per unit area, the irradiance of the sensor.

Assuming the characteristics of a point source and the validity of the thin lens approximation, we show that if the sensor is positioned in the focal plane of the lens (at $z_s = f$), the area of the optical image is inversely proportional to the square of the distance between light source and sensor. This yields that the reduction in the amount of light on the sensor cancels out the reduction in image size. Hence the irradiance of the image remains constant when z is varied.

We assume that the quantum efficiency, as expressed by the sensor response function F, depends on the irradiance. Under the above assumptions, the irradiance is constant over the area of the image. Thus, the normalized sensor response of the detector positioned in the focal plane is identical for any distance z.

Even though the conditions in the actual experiments deviate significantly from the assumptions defined above, we find that responsivity curves at various distances for sDSSC (Fig. 3a), PbS photoconductors (Fig. 4a) and amorphous silicon (Fig. S7.1) intersect close to the focal length of the used lens.

S6 - Modelling the photoresponse of partially illuminated photoconductors

We model a photoconductor device as an infinitesimally fine network of light dependent resistors. The current flow is governed by the Ohm's law, i.e. $U = R \cdot I$.

The goal is to derive a continuous model of the network by a limit process for mesh sizes Δx approaching zero (Fig. S6.1). The result of the limit process is an elliptic partial differential equation that describes the current flow within a photoconductor.

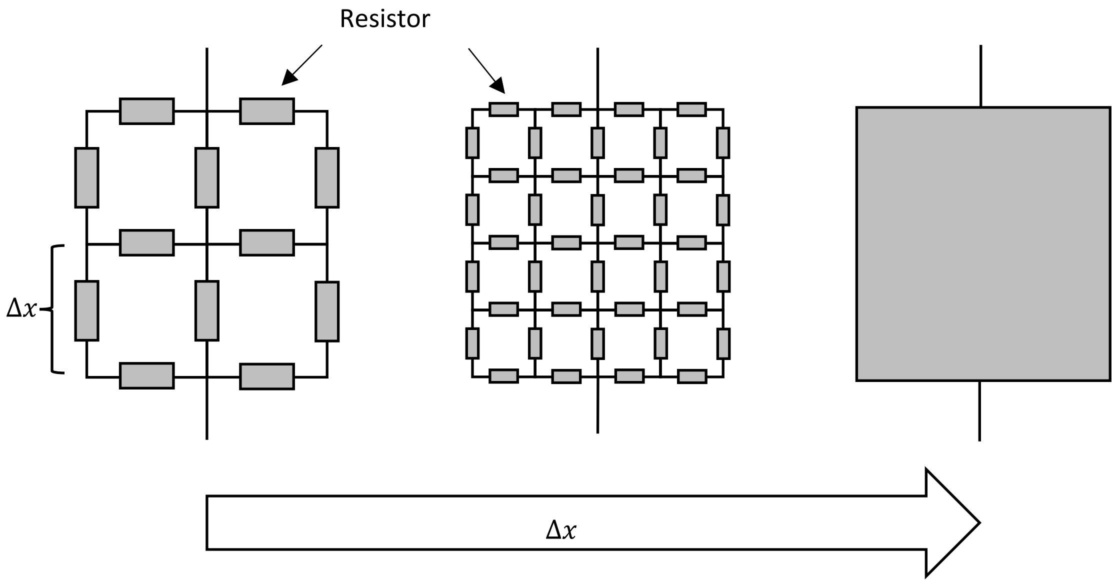

Fig. S6.1 Visualization of a limit process of electricity network. The photoconductor is modeled as a coarse electricity network.

The continuous model can be derived as follows:

We can interpret the solution of the resistor network as the solution of a two-dimensional finite-volume scheme. Each arc of the network should have the same length Δx. We assume that the values of a node correspond the value of a cell (see Fig. S5.2). For more details see: *Peter Schillen - Modelling and Control of Balance Laws with Applications to Networks – Dr. Hut - ISBN* 9783843922159 – *Section 3.6*. The voltage is given by the finite difference of the potential φ, e.g. $U_N = (\varphi_{i,j+1} - \varphi_{i,j})$.

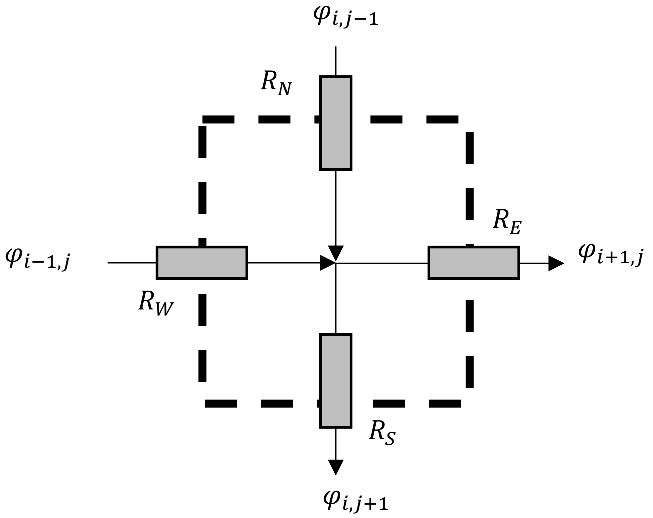

Fig. S6.2 Scheme of a node in the resistor network

Kirchhoff's current laws and Ohm's law yield the following equation

$$0 = \frac{I_S - I_N}{\Delta x} + \frac{I_E - I_W}{\Delta x} = \frac{(\varphi_{i,j+1} - \varphi_{i,j})R_N - (\varphi_{i,j} - \varphi_{i,j-1})R_S}{\Delta x^2 R_S R_N} + \frac{(\varphi_{i,j+1} - \varphi_{i,j})R_W - (\varphi_{i,j} - \varphi_{i,j-1})R_E}{\Delta x^2 R_E R_W} \qquad (S6-1)$$

For $\Delta x \rightarrow 0$ the finite differences coincide with its derivatives.

$$0 = \frac{\partial_{xx}\varphi R(x,y) - \partial_x\varphi\partial_x R(x,y)}{R(x,y)^2} + \frac{\partial_{yy}\varphi R(x,y) - \partial_y\varphi\partial_y R(x,y)}{R(x,y)^2} \qquad (S6-2)$$

It is necessary to assume that R is weakly differentiable.

Finally, the limit yields a continuous model based on a partial differential equation

$$\nabla \cdot \left(\frac{\nabla\varphi}{R(x,y)}\right) = 0, \qquad (S6-3)$$

for all $x \in \Omega$, where Ω is the cell domain. Note that Kirchhoff's voltage law is already fulfilled by the fundamental theorem of calculus.

Additionally, boundary conditions are required for unique solutions: The first case is that the boundary of a cell is connected to a voltage source. That means that the potential on the boundaries is described by a known function φ_0, i.e.

$$\varphi(x) = \varphi_0(x)\ \forall x \in \partial\Omega. \qquad (S6-4)$$

The boundary of the cell domain Ω is denoted by $\partial\Omega$.

The second case is that there is no electrical connection between the cell boundary and a voltage source, e.g. isolation by air. Then the boundary condition is given by

$$\nabla\varphi(x) \cdot n = 0\ \forall x \in \partial\Omega, \qquad (S6-5)$$

where n is the outgoing normal of the domain Ω. Note that these two cases of boundary conditions can be mixed. An example is given in Fig. S5.3. The cell has two electrodes with a voltage of U_0 and two isolated edges.

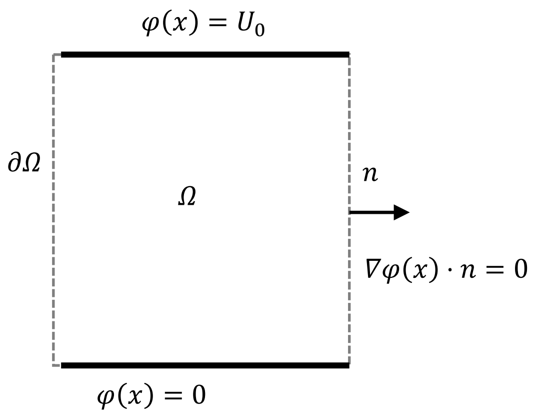

Fig. S6.3 Example of a cell domain Ω. The continuous edge (top and bottom) corresponds to the cell electrodes with a voltage of U_0. The dashed line (left and right) indicates the part without any voltage source.

Analytical solution for the one-dimensional case:

For the one-dimensional case $\partial_x\left(\frac{\partial_x\varphi}{R(x)}\right) = 0$ we can solve the equation analytically. First, we express the resistance in the form of conductivity, i.e. $R(x) = \frac{1}{\sigma(x)}$. By integrating over x we get:

$$\varphi(x) = c_0 \int_0^x R(s)\, ds + c_1 \qquad (S6-6)$$

We consider an area of length b, with boundary values $\varphi(0) = 0, \varphi(b) = U_0$, hence yielding a voltage of U_0. With the boundary values, it follows that, $c_1 = 0,\ c_0 = U_0 \frac{1}{\int_0^b R(s)ds}$. As one can see, c_0 is the current of the system. We assume that the conductivity of a photoconductor device changes with the light intensity and the geometry of the cell.

We consider a one-dimensional photoconductor. If the length is fixed, the conductivity only depends on the light intensity. The dark conductivity σ_d changes upon illumination by $\Delta\sigma = p \cdot$

E, $\Delta\sigma$ hence depending linearly on the irradiance E. In Fig. S6.4 a one-dimensional photoconductor is shown schematically.

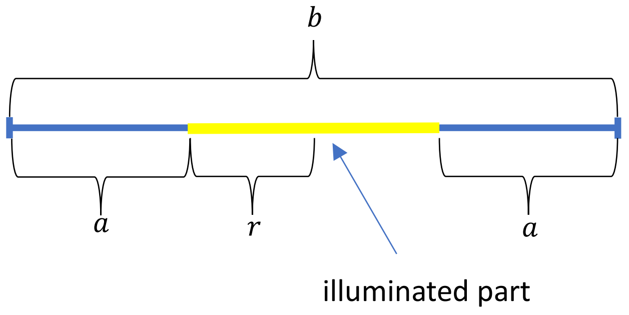

Fig. S6.4 Scheme of a partially illuminated one-dimensional photoconductor.

Now we can compute $\int_0^b R(s)\,ds = \int_0^b \frac{1}{\sigma(s)}ds = \int_0^a \frac{1}{\sigma_d}ds + \int_a^{a+2r}\frac{1}{\sigma_d+pE}ds + \int_{a+2r}^{2(a+r)}\frac{1}{\sigma_d}ds =$ $2r\frac{1}{\sigma_d+pE} + 2a\frac{1}{\sigma_d} = \frac{2r\sigma_d+2a(\sigma_d+pE)}{\sigma_d(\sigma_d+pE)}$. Now we have $c_0 = \frac{\sigma_d(\sigma_d+pE)}{2r\sigma_d+2a(\sigma_d+pE)}U_0$.

Theorem: $c_0(E)$ is affine linear for $a = 0$ (i.e. the sensor is fully and uniformly illuminated) and non-linear for $a > 0$.

Proof: We derive $c_0(E) = \frac{\sigma_d(\sigma_d+pE)}{2r\sigma_d+2a(\sigma_d+pE)}U_0$ w.r.t. E and get $\frac{d}{dE}c_0(E) = \frac{p\sigma_d^2 r}{2(a(pE+\sigma_d)+\sigma_d r)^2}U_0$.

Trivially $\frac{d}{dE}c_0(E)$ does not depend on E for $a = 0$, otherwise for $a \neq 0$ it does. This proves that $c_0(E)$ is affine linear for $a = 0$.

q.e.d.

S7 – Long-range measurements over 72 m using amorphous silicon-based FIP detectors

Long-range measurements up to 72 m are performed using an amorphous silicon based FIP detector. The distance to a modulated LED emitting at 660 nm is determined using the FIP technique. The LED light is collected by a 95 mm diameter commercial lens (Walimex pro 500 mm), which directs the converging beam towards the sensors. A stack of two semitransparent amorphous silicon solar cells (40 x 40 mm active area, 500 nm i-layer thickness, custommade by Solems S. A.) operating at short circuit is used as detector. To cover distances beyond 36 m, a 20 x 30 cm large mirror is used as reflector, allowing for measurements up to 72 m.

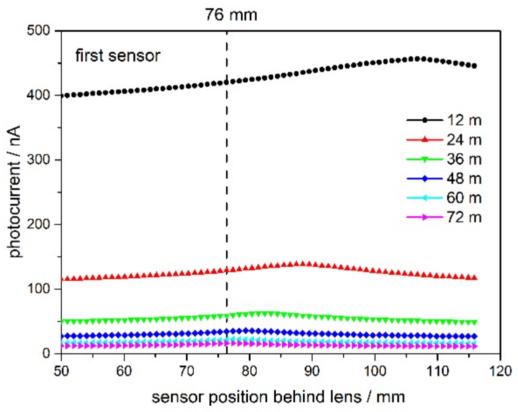

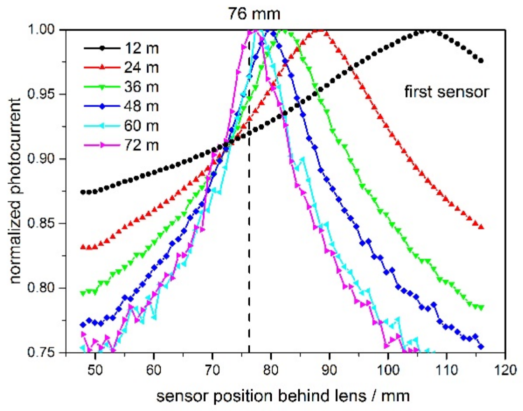

Fig. S7.1 Short circuit photocurrent of the first sensor at various positions behind the lens for several LED distances ranging from 12 m to 72 m. The dashed lines indicate the sensor position used for the quotient determination.

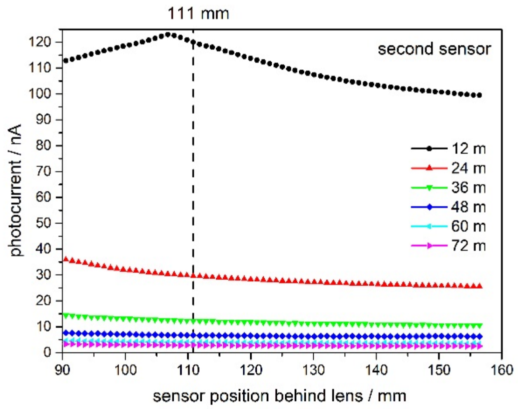

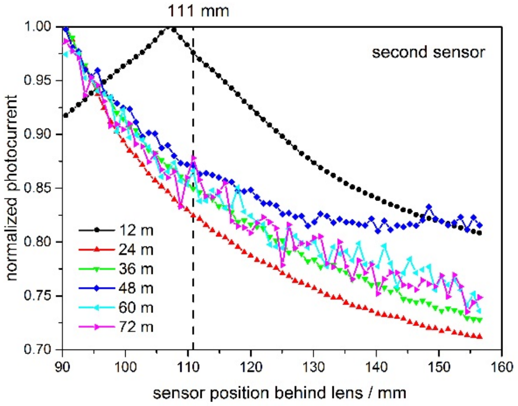

Fig. S7.2 Short circuit photocurrent of the second sensor at various positions behind the lens for several LED distances ranging from 12 m to 72 m. The light passes through the first sensor before impinging on the second. The dashed lines indicate the sensor position used for the quotient determination.

Figures S7.1 and S7.2 show the signals of both sensors in the detector stack. The data is collected for each LED distance by moving the stack behind the lens on the optical axis. The photocurrent in the nano Ampere regime is measured using a Fourier transform technique.

In order to perform distance measurements in the range between 12 m and 72 m, the first sensor is positioned 76 mm and the second 110 mm from the back of the lens. Calculating the ratio of

the first sensor signal to the second sensor at these positions for various LED distances yields the calibration curve depicted in Fig. S7.3. The quotient of the sensor signals is shown to monotonically increase over the entire measurement range, making it possible to assign a single quotient value to any distance up to 72 m.

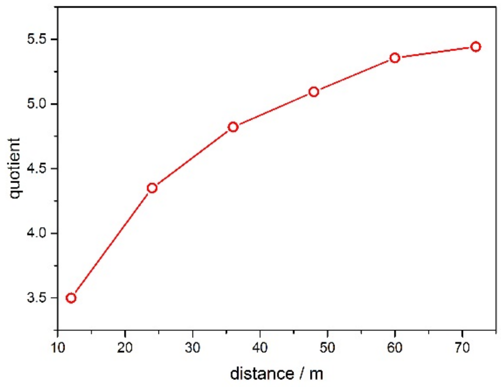

Fig. S7.3 Calibration curve for distance measurements generated by calculating the quotient of the two sensor signals at 12m, 24 m, 36 m, 48 m, 60 m and 72 m. The line acts as guide to the eye.
